# Atypical Teratoid Rhabdoid Tumor: Current Therapy and Future Directions

**DOI:** 10.3389/fonc.2012.00114

**Published:** 2012-09-12

**Authors:** Kevin F. Ginn, Amar Gajjar

**Affiliations:** ^1^Division of Neuro-Oncology, St. Jude Children’s Research HospitalMemphis, TN, USA

**Keywords:** ATRT, pediatric brain tumors, cyclin D1, aurora kinase, insulin-like growth factor, tyrosine kinase inhibitors

## Abstract

Atypical teratoid rhabdoid tumors (ATRTs) are rare central nervous system tumors that comprise approximately 1–2% of all pediatric brain tumors; however, in patients less than 3 years of age this tumor accounts for up to 20% of cases. ATRT is characterized by loss of the long arm of chromosome 22 which results in loss of the *hSNF5/INI-1* gene. INI1, a member of the SWI/SNF chromatin remodeling complex, is important in maintenance of the mitotic spindle and cell cycle control. Overall survival in ATRT is poor with median survival around 17 months. Radiation is an effective component of therapy but is avoided in patients younger than 3 years of age due to long term neurocognitive sequelae. Most long term survivors undergo radiation therapy as a part of their upfront or salvage therapy, and there is a suggestion that sequencing the radiation earlier in therapy may improve outcome. There is no standard curative chemotherapeutic regimen, but anecdotal reports advocate the use of intensive therapy with alkylating agents, high-dose methotrexate, or therapy that includes high-dose chemotherapy with stem cell rescue. Due to the rarity of this tumor and the lack of randomized controlled trials it has been challenging to define optimal therapy and advance treatment. Recent laboratory investigations have identified aberrant function and/or regulation of cyclin D1, aurora kinase, and insulin-like growth factor pathways in ATRT. There has been significant interest in identifying and testing therapeutic agents that target these pathways.

## Introduction

Primary central nervous system (CNS) atypical teratoid rhabdoid tumors (ATRTs) were identified as a separate entity relatively recently, as recognized by their addition to the World Health Organization (WHO) classification of tumors in 1993 (Biernat, 2000; Radner et al., 2002). The first reported cases appeared as multiple case reports in the late 1980’s and early 1990’s that described patients, for the first time, with isolated CNS ATRTs (Bonnin et al., 1984; Biggs et al., 1987; Ho et al., 1990; Chou and Anderson, 1991; Agranovich et al., 1992; Satoh et al., 1993). Previously reported cases of CNS rhabdoid tumors were often associated with malignant rhabdoid tumors (MRT) of the kidney, which had been identified a decade earlier upon central review of Wilms’ tumors treated in a collaborative group (Beckwith and Palmer, 1978). This group noted that certain patients had tumors with different pathologic features and that these features were associated with significantly worse outcomes. Similarly, ATRTs were often categorized with primitive neuroectodermal tumors (PNETs), along with medulloblastoma, due to histologic similarities, but they are now separated from other embryonal tumors by the presence of rhabdoid cells and specific immunohistochemistry (Biggs et al., 1987; Lefkowitz et al., 1987; Ho et al., 2000; Bikowska et al., 2011). Like rhabdoid tumors of the kidney, CNS ATRTs are also associated with significantly worse overall survival than other embryonal tumors.

A recent review of the Surveillance, Epidemiology, and End Results (SEER) database from 1973 to 2008 estimated overall survival for ATRT patients at 10 months, and notably the year of diagnosis had no effect on survival, showing that little progress has been made since ATRTs were first identified (Buscariollo et al., 2011). Another review from the German HIT database from 1988 to 2004 showed 77% of patients with ATRT (43/56) died of disease [3-year event-free survival (EFS), 13 ± 5%; overall survival (OS), 22 ± 6%; von Hoff et al., 2011]. Investigators from The Hospital for Sick Children compiled data from four patients with ATRT at their institution with a retrospective review of 143 cases reported in the literature from 1995 through 2007 and found a median overall survival of 17.3 months (Athale et al., 2009). Overall, patients usually succumb to their disease between 6 months and 1 year from diagnosis. Survival is especially poor for patients with metastatic disease, which one registry found was present in around 20% of patients at diagnosis (Hilden et al., 2004).

ATRT accounts for 1–2% of CNS tumors in children of all ages, but 10–20% of tumors in patients less than 3 years old (Hilden et al., 2004; Tekautz et al., 2005; Biegel, 2006), who also tend to present with infratentorial tumors (70%; Rorke et al., 1996). The higher frequency of ATRT in patients less than 3 years old complicates therapy due to avoidance of radiation therapy (RT) in this age group, and these patients have shorter survival than older patients with the same tumor. Delayed radiation can affect prognosis. Multiple chemotherapy approaches to defer radiation have been attempted, yet overall survival remains dismal.

In 1995, Rorke et al. (1995) published one of the earliest and largest case series of 32 infants with CNS ATRT. They found a 1.9:1 male-to-female ratio, a median age at diagnosis of 17 months, and an association with chromosome 22 abnormalities. In subsequent years, investigation into associated chromosome 22 abnormalities led to the identification of *h*SNF5/INI1 gene mutations that are now the defining feature of this entity and the main target of current research to identify new therapeutic approaches (Versteege et al., 1998).

This aggressive tumor remains a significant challenge in pediatric neuro-oncology, and new therapeutic approaches are desperately needed. This review will summarize the published therapies to date and focus on recent basic science and translational studies as new potential targets are being identified in the laboratory and there is a need to push for advances in therapy that may lead to increased survival for patients with this devastating tumor.

## Chemotherapy

Given the rarity of ATRTs and the variety of treatment regimens used to date, no standard therapeutic approach has been established. Published case series often include patients treated with multiple therapeutic approaches, making standardization of therapy difficult. Table 1 summarizes some of the larger clinical trials that included ATRT patients or that were designed specifically for ATRT. Early complexity determining the best therapeutic approach was evident in the reported proceedings of the tumor board of The Children’s Hospital of Philadelphia (CHOP) in 1991 (Perilongo et al., 1991). The group described a 5-year-old patient with a CNS primary rhabdoid tumor and detailed their consideration of therapeutic approaches including review of children with rhabdoid tumors of the kidney. Ultimately, they opted to treat with 3600 cGy craniospinal radiation with an unspecified boost dose to the primary site along with chemotherapy that was currently in use on the infant brain tumor therapeutic trials, including cisplatin, cyclophosphamide, vincristine, and etoposide.

**Table 1:** **Published reviews and clinical trials that included or were specific to atypical teratoid rhabdoid tumors**.

Study time period	*n* of patients	Age	Type of study	Chemotherapy	Radiation	Survival
Tekautz et al. (2005)	31	22 patients <3 years (median 1 year) 9 patients ≥3 years (median 3.9 years)	Retrospective review	Multiple regimens	<3 years 2 local, 1 CSI + boost	<3 years (estimates) 2-year EFS 11 ± 6% 2-year OS 17 ± 8%						
July 1984–June 2003					≥3 years 7 patients CSI + boost	≥3 years (estimates) 2-year EFS 78 ± 14% 2-year OS 89 ± 11%
						
Geyer et al. (2005)	28	12 patients aged 0–11 months.	Phase II/III	Induction A (*n* = 16) VCR/CDDP/CTX/VP	2 patients prior to progression (1 focal, 1 CSI)	1-year EFS 32 ± 9% 2-year EFS 14 ± 7%
CCG9921		6 patients aged 12–17 months.		Induction B (*n* = 12) VCR/CDDP/IFOS/VP		5-year EFS 14 ± 7% 5-year OS 29 ± 9%
April 1993–June 1997		10 patients aged 18–36 months.		Maintenance VCR/CDDP/CTX/VP	
Douglas Strother [personal communication 2011] POG9233/34 1992–1998	33	All patients <3 years	Phase III	CTX/VCR/CDDP/VP Standard versus dose-intensified	None	5 year OS 0% Median survival 6.7 months.
Lafay-Cousin et al. (2012)	50	Median age 16.7 months.	Retrospective review	Multiple regimens	21 patients at some point during therapy	2-year OS 36.4 ± 7.7%
		17 patients aged <12 months.		22 conventional		
		21 patients aged 12–36 months.		18 high-dose chemo	6 patients at time of relapse	
1995–2007		12 patients >36 months.			
Chi et al. (2009)	20	Median 26 months (2.4 month–9.5 years)	Phase II	Modified IRS-III	54 Gy focal (*n* = 11)	2-year PFS 53 ± 13%
					36 Gy CSI + boost (*n* = 4)	2-year OS 70± 10%
2004–2006

*CDDP, cisplatin; CSI, craniospinal radiation; CTX, cyclophosphamide; EFS, event free survival; IFOS, ifosfamide; IRS, Intergroup rhabdomyosarcoma study; OS, overall survival; PFS, progress free survival; VCR, vincristine; VP, etoposide*.

In response to the report from CHOP, Weinblatt and Kochen (1992) from Cornell submitted a letter describing a patient they treated in 1985 with a primary CNS rhabdoid tumor with gross total resection (GTR), 4140 cGy focal radiation, and intensive chemotherapy as per the Intergroup Rhabdomyosarcoma III (IRS-III) therapy, including weekly vincristine during radiation, actinomycin-D, doxorubicin, and triple intrathecal chemotherapy with hydrocortisone, methotrexate, and cytosine arabinoside. An additional three cases successfully treated with IRS-III were then reported in 1995 by Ohio State University (Olson et al., 1995). This approach was justified because ATRT was thought to be similar to parameningeal rhabdomyosarcomas, requiring more aggressive therapy, and regimen 36 was chosen because it was intensive chemotherapy that was easily adaptable to radiation and triple intrathecal chemotherapy. This group also summarized the 18 cases of primary CNS MRTs previously reported in the literature to date, showing the varied approaches to therapy, and the need for a more standardized approach.

Dana-Farber Cancer Institute (DFCI) decided to return to the original early reports of success with IRS-III-based regimens to treat two new ATRT patients and two with recurrent disease between December 1999 and April 2002 due to unpublished reports of failures with modifications from the original therapy (Zimmerman et al., 2005). DFCI modifications included focal stereotactic RT without a craniospinal dose for patients less than 3 years old, substitution of dacarbazine with temozolomide, and addition of dexrazoxane for cardioprotection in higher cumulative doses of doxorubicin. All four patients were alive at the time of reporting at a median 44.5 months after diagnosis and a median 26.5 months after recurrence. A later update from the group at DFCI stated that three of the four were alive at a median 6.5 years after completion of therapy. DFCI then proceeded with a phase II study between February 2004 and September 2006 with a modified IRS-III protocol and treated 20 patients with ATRT (Chi et al., 2009). Eight of the 20 patients had relapses by the time of publication, giving a 1-year progression-free survival (PFS) rate of 70 ± 10% and OS of 75 ± 10% and 2-year PFS of 53 ± 13% and OS 70% ± 10%. Univariate analysis showed that PFS and OS were significantly influenced by the extent of resection. OS was also affected by tumor location, and patients with posterior fossa tumors had better survival. The reported PFS and OS were significantly better than those seen in other clinical trials but, due to small numbers, it was impossible to make comparisons to determine why there was such an improvement. This report did, however, point to improved survival with intensified chemotherapy that included intrathecal administration along with focal radiation for those patients younger than 3 years old. It would also have been useful with larger numbers to separate the overall survival data based upon age, as others have shown that survival is improved for older patients.

Modified IRS-III therapies include intrathecal chemotherapy as well as multiagent chemotherapy and focal radiation in patients who have non-metastatic disease. Intrathecal chemotherapy may have potential benefit as an additional means to avoid radiation or to intensify therapy in patients who are not candidates for craniospinal radiation. A meta-analysis by Athale et al. (2009) showed that even without GTR, patients who received multiagent chemotherapy fared better, but this effect was most prominent in those less than 3 years old who did not get radiation. Without radiation, intrathecal chemotherapy also made a significant difference in overall survival (OS 10.5 months versus 6.5 months, *p* = 0.011).

An additional approach to early therapy of ATRT was explored as these patients were included in national infant brain tumor clinical trials. The North American Children’s Cancer Group from April 1993 through June 1997 enrolled 299 children less than 3 years old with multiple tumor types on protocol CCG9921 (Geyer et al., 2005). This regimen included two induction courses with ifosfamide or cyclophosphamide along with vincristine, cisplatin, and etoposide. Induction was followed by maintenance with vincristine, etoposide, carboplatin, and cyclophosphamide. The study included 28 rhabdoid tumors (9.4% of patients enrolled), and 24 of those had treatment failures. The 1-year and 5-year EFS rates were 32 ± 9% and 14 ± 7%, respectively, and the 5-year OS was 29 ± 9%. Interpretation of factors associated with prognosis was difficult due to the small numbers in this group. During the same period, the Pediatric Oncology Group was investigating the use of standard versus dose-intensified chemotherapy to delay radiation in young children on POG 9233/34 (Baby POG 2). The study enrolled 36 patients with ATRT. Chemotherapy included cyclophosphamide, vincristine, cisplatin, and etoposide. Patients on the dose-intensified arm had better responses, but all patients with rhabdoid tumors ultimately died, with a median survival of 6.7 months (personal communication, Douglas Strother).

Researchers from St. Jude Children’s Research Hospital in 2005 reported a retrospective review of 31 patients with ATRT treated between 1984 and 2003 (Tekautz et al., 2005). As expected, the patients were treated with multiple different approaches over the 20-year period, but overall they determined that outcomes were better for patients who were older than 3 years at diagnosis and those who had received craniospinal radiation and high-dose alkylator-based chemotherapy up front with a 2-year OS of 89 ± 11%. Five of the nine patients older than 3 years were alive without recurrence at the time of publication at a median of 2.2 years, and it is important to note that seven of those patients had craniospinal radiation. In contrast, only four of 22 patients less than three were without relapse. One of the four died of a surgical complication; of the remaining three, two received RT.

The Canadian Pediatric Brain Tumor Consortium recently published a retrospective review of patients with ATRT treated from 1995 through 2007 (Lafay-Cousin et al., 2012). They identified 50 patients, and although there were multiple different therapies during the period, they were able to make several conclusions from their analysis. As expected, the prognosis was much worse for patients younger than 12 months. Patients with GTR had better survival at 2 years at 60 ± 12.6% versus 21.7 ± 8.5% (*p* = 0.03). They did not find a significant difference in survival related to radiation, but they did find that patients treated with high-dose chemotherapy (HDC) had better 2-year OS at 47.9 ± 12.1% versus 27.3 ± 9.5% (*p* = 0.036).

There seems to be improved survival for those patients treated on IRS-III based *de novo* therapy and high-dose alkylating agent compared to other chemotherapy approaches although numbers are small in all series and it is difficult to separate the role the modifications of the IRS-III regimen. As stated earlier, intrathecal chemotherapy has been associated with improved survival in those patients that did not receive radiation. In the IRS-III based regimen, the combination of intrathecal chemotherapy with focal radiation in those patients less than 3 years of age may be an effective strategy for disease control, but intrathecal chemotherapy in those patients older than 3 years may not be needed since craniospinal radiation is an acceptable option. It is clear that intensive systemic chemotherapy alone as a method to avoid radiation is not effective in ATRT and radiation must be considered much earlier in therapy than previously thought appropriate, but with continued respect for long term effects.

Since 2008, the Children’s Oncology Group (COG) has been enrolling patients with ATRT on a clinical trial that incorporates induction chemotherapy with high-dose methotrexate, focal radiation in patients as young as 6 months for infratentorial tumors and 12 months for supratentorial tumors, and three cycles of consolidation with thiotepa and carboplatin with autologous stem cell support. This therapeutic protocol thus incorporates focal radiation in younger patients than on previous COG protocols. High-dose methotrexate was included based on data from the Head Start (HS) protocols discussed later and the three cycles of consolidation are based on CCG 99703 for which data has yet to be published. The study was closed for 1 year due to the toxic death of one patient but has been reopened with amendments concerning pulmonary toxicity monitoring and increased time between consolidation courses. Enrollment is ongoing, and the accrual rate is as expected for this rare tumor.

## Radiation

Previous studies that aimed to avoid or delay radiation in ATRT patients less than 3 years old were associated with a very poor prognosis, and some clinical trials now incorporate focal radiation in much younger patients than previously thought appropriate. Radiation has been associated with improved survival in ATRT, especially in patients who have craniospinal radiation with a focal boost to the tumor bed. There are two published series that were intended to address the issue of radiation in patients with ATRT, and both support radiation as soon after surgery as possible. Other case series in the literature are not included here, but most show outcomes are better in patients who received radiation as noted previously.

St. Jude researchers reviewed 31 ATRT patients treated from 1987 through 2007, which also included some patients previously reported by Tekautz et al. (2005), and aimed to evaluate patterns of failure and local control with radiation (Pai Panandiker et al., 2011). Again, patients had varying chemotherapy regimens and extent of resection, but all were treated with focal radiation alone or with the addition of craniospinal irradiation (CSI). At a median follow up of 48 months, the PFS was 32.2 ± 10%, and OS was 53.5 ± 10%. Using a Cox regression model, they showed that patients with a GTR and stable disease before RT were less likely to experience an adverse event, while conversely patients with delayed RT were more likely to experience an event. Delayed RT was defined as ≥1 month from surgery. Overall survival was affected only by disease progression before RT in their analysis. Metastatic disease at presentation did not significantly affect PFS or OS. Those with less than GTR had a local failure rate of 53.3 ± 14% at 4 years, and those with GTR had a local failure rate of 17.9 ± 10%. Of those who had immediate postoperative CSI, 29% (2/7) experienced local failure compared with 58% (7/12) of those who had delayed postoperative CSI. The six patients younger than 3 years who were alive at last follow up before publication all had focal RT.

Seventeen patients with ATRT were treated at Taipei Veterans General Hospital in Taiwan between January 1990 and December 2003 (Chen et al., 2006). Fifteen of 17 patients had the standard study regimen of CSI ranging from 2550 to 3600 cGy for prophylaxis or 3600 cGy plus a focal boost up to 4400 cGy for spinal seeding. The total primary dose ranged from 4860 to 5600 cGy. Two of 17 patients had whole brain radiation with a focal boost. Patients received varying approaches to chemotherapy before, during, or after radiation, with the most common chemotherapy including vinblastine, ifosfamide, and cisplatin. Nine of the 17 received intrathecal chemotherapy with either methotrexate or nimustine. The median OS was 17 months and 14 (82%) had relapses with median time from relapse to death of 6 months. The group found no difference in OS or failure-free survival between doses more or less than 5000 cGy. Multivariate analysis showed a significant relationship between the interval between surgery and RT (*p* = 0.031) and the time to radiation completion (*p* = 0.047). When evaluating the six surviving patients at the time of publication, three of whom had no evidence of disease, and one of those who had never received any chemotherapy, they noted that these patients tended to be older, had a GTR, and completed CSI with a focal boost.

## High-Dose Chemotherapy

Table 2 summarizes patients reported in the literature who have had HDC as a significant element of their therapy for ATRT. For each study listed in the table, only the patients who proceeded to the HDC phase of therapy are shown. HDC with autologous stem cell rescue has been used not only as salvage therapy for patients with relapsed disease, but also as a method of intensifying initial chemotherapy to delay irradiation in young patients. St. Jude researchers retrospectively reviewed 27 cases of recurrent malignant brain tumors treated with HDC with autologous stem cell rescue between March 1989 and May 2004. Of the 27 patients treated with multiple chemotherapy regimens, only two had a diagnosis of ATRT. The estimated 5-year PFS for all embryonal tumors in patients less than 3 years old at the time of diagnosis was 66.7 ± 22.2% versus 7.1 ± 4.9% for those 3 years or older. This difference was likely because the patients younger than three were able to be salvaged with HDC and radiation, whereas those older than three had previously received radiation so it was not an option for salvage. One patient with ATRT who was 1 year old was treated with chemotherapy alone at diagnosis and had residual tumor with positive cerebrospinal cytology before HDC. Salvage therapy included high-dose cyclophosphamide and topotecan; disease persisted after HDC. Time to progression was 39 days, and the patient died of disease 70 days after salvage therapy. The other patient with ATRT, also 1 year old, received chemotherapy alone at diagnosis and had no evidence of disease at time of salvage therapy, which included focal RT and HDC with cyclophosphamide and topotecan. At the time of publication, he remained alive with no evidence of disease after more than 862 days (Shih et al., 2008). Although it is difficult to generalize this information, the results are as would be expected, in that patients with minimal to no detectable disease at the time of HDC are more likely to have prolonged survival.

**Table 2:** **Cases reported in the literature in which the main focus of therapy for ATRT was high-dose chemotherapy with autologous stem cell rescue**.

Published report	Type of study	Age (month)	Pre-HDCT treatment	Disease status prior to HDCT	RT	HDCT	Response to HDCT	Relapse data	Adjuvant therapy	Survival outcome
Shih et al. (2008)	Recurrent/refractory	12	Chemotherapy	Nodule, CSF+	None	CTX/TOPO	PD	None	None	70 days from day +1, DOD
	March 1989–May 2004	13	Chemotherapy	NED	Local prior to HDCT	CTX/TOPO	NED	None	None	862+ days from day + 1, alive, NED
Gardner et al. (2008)	*De novo* therapy, Head Start I protocol, Induction with CDDP, VP-16, CPM, and VCR	45	HS-I induction courses x3 (no VP during cycle 3)	NED	None	CARBO/THIO/VP	NED	Local	None	10.5 month, DOD
	Open 1992–1997 (4 patients did not proceed to consolidation due to progression)	44	None	GTR, M3	None	CARBO/THIO/VP	CR	LMD, right frontal lobe	None	10.5 month, DOD
	*De novo* therapy, Head Start II protocol, Induction as per, HS-I + HD-MTX	45	HS-II induction courses x5 (no MTX)	CR	None	CARBO/THIO/VP	CR	N/A	None	Alive, 67+ month, NED
	Open 1997–2002 (2 patients did not proceed to consolidation due to progression)	23	HS-II induction courses x5	CR	None	CARBO/THIO/VP	CR	N/A	None	Alive, 42+ month, NED
		52	HS-II induction courses x5 (no CDDP during course 5 due to ototoxicity)	CR	CSI after HDCT before relapse	CARBO/THIO/VP	CR	Local, LMD, PF, SC	None	11.5 month, DOD
		5	HS-II induction courses x5	SD	Involved field after relapse	CARBO/THIO/VP	SD	Local	None	36 month, died of 2nd leukemia
		48	HS-II induction courses x5	CR	None	CARBO/THIO/VP	CR	None	None	Alive, 54+ month, NED
Fidani et al. (2009)	*De novo* therapy, CECAT replaced with additional courses of ICE after 3 out of 4 progressed (5 of 8 progressed during primary chemotherapy)	29	ICE x2, CECAT x2	CR	Yes	VP/THIO/CTX	CR	NONE	None	Alive, 105+ month, NED
		42	ICE x2, CECAT x2	PD	Yes at PD	VP/THIO/CTX (After PD)	NED	Relapse after CECAT, then to surgery, HDC, RT	None	Alive, 101+ month, NED
		91	ICE x4	CR	Yes	VP/THIO/CTX	PD	Relpase then TVDx6/TMZ	None	AWD, 38+ month
		44	ICE x2	PR	On therapy	On therapy	N/A	N/A	None	AWD, 5+ month
Finkelstein-Shechter et al. (2010)	Retrospective review of patients treated 2003–2008	43	5 patients had 3 cycles VP-16, CTX, CDDP, VCR; 1 pt treated with IRS-III	Not reported	None	3 cycles of CARBO/THIO	Not reported	N/A	None	Alive, 64 month, NED
		11			None			N/A	TAM	Alive, 51 month, NED
		31			Focal			N/A	None	Alive, 52 month, NED
		40			Focal during first 2 chemo cycles			Relapse 16 month	None	DOD
		39			None			Relapse 8 month	TAM	DOD
		28			None			N/A	Intraventricular TOPO, TAM-ATRA	Alive, 23 month, NED
Nicolaides et al. (2010)	*De novo* therapy May 1997–January 2007 (3 patients died of disease prior to HDCT)	49	MTX, CTX,VP, CDDP, VCR (HSII)	CR	Local	CARBO/VP/THIO	Not reported	N/A	None	Alive, 78 month, NED
		46	T-IT, CDDP, VP, VCR, AD, IFOS, CTX	CR	None	CARBO/VP/MELPH/CTX	Not reported	Disseminated at 2 month	None	DOD, 10 month
		15	MTX, CTX, VP, CDDP, VCR, IT-ARAC	CR	None	CARBO/THIO/VP	Not reported	Disseminated at 3 month	None	DOD, 10 month
		6	MTX, CTX, VP, CDDP, VCR (2/3 with MTX)	CR	None	THIO/TOPO/VP	Not reported	N/A	None	Alive, 98 month, NED
		9	CDDP, VP, VCR, CTX	CR	None	CARBO/THIO	Not reported	Distant relapse	None	DOD, 13 month
		33	CDDP, VP, VCR, CTX	CR	None	CARBO/THIO	Not reported	N/A	None	AWD, 19 month
Park et al. (2012)	*De novo* therapy, phase I/II prospective trail tandem HDCT	4	Alternating CECV and CEIV x6 cycles	PR	None	CARBO/THIO/VP then CTX/MELPH	Not reported	Progression at 13 month	None	DOD, 15 month
	September 2005–March 2010 2 patients did not proceed to HDCT due to progression, 1 pt died of sepsis during induction	9	Alternating CECV and CEIV x6 cycles	PD	CSI/boost (after HDCT)	CARBO/THIO/VP then CTX/MELPH (after PD, surgery, then HDCT followed with RT)	Not reported	Progression at 8 month then surgery, HDC1/2, then RT	None	Alive, 16+ month, NED
		11	Alternating CECV and CEIV x6 cycles	CR	Local	CARBO/THIO/VP then CTX/MELPH (after relapse)	Not reported	Relapse at 6 month then repeat surgery, RT, and HDCT1/2	None	Alive, 50+ month, NED
		12	Alternating CECV and CEIV x6 cycles	CR	CSI/boost (after progression)	CARBO/THIO/VP then CTX/MELPH	Not reported	Relapse at 21 month post HDCT then Sx, CECV/CEIV, RT	None	Alive, 70+ month, NED
		15	Alternating CECV and CEIV x4 cycles	PR	CSI/boost (after progression)	CARBO/THIO/VP then CTX/MELPH (after progression)	Not reported	Progression at 4 month then Sx, RT, HDCT1/2, progression, CTx	None	AWD, 19+ month
		28	Alternating CECV and CEIV x6 cycles	PR	CSI/boost after HDCT	CARBO/THIO/VP then CTX/MELPH	Not reported	N/A	None	Alive, 20+ month, NED

*AD, actinomycin D; ATRA, all-trans retinoic acid; AWD, alive with disease; CARBO, carboplatin; CDDP, cisplatin; CECAT, cyclophosphamide, etoposide, carboplatin, thiotepa; CECV, cisplatin, etoposide, cyclophosphamide, vincristine; CEIV, carboplatin, etoposide, ifosfamide, vincristine; CR, complete response; CSF, cerebrospinal fluid; CSI, craniospinal radiation; CTX, cyclophosphamide; DOD, died of disease; GTR, gross total resection; HDCT, high-dose chemotherapy; ICE, ifosfamide, carboplatin, etoposide; IFOS, ifosfamide; IT-ARAC, intrathecal cytosine arabinoside; LMD, leptomeningeal disease; MELPH, melphalan; MTX, methotrexate; NED, no evidence of disease; PD, progressive disease; PF, posterior fossa; RT, radiation therapy; SC, spinal cord; SD, stable disease; TAM, tamoxifen; THIO, thiotepa; T-IT, triple intrathecal chemotherapy; TOPO, topotecan; TVD, topotecan, vincristine, doxorubicin; VCR, vincristine; VP, etoposide*.

Gardner et al. (2008) reported their experience treating patients with CNS ATRT enrolled on the HS I and II protocols. Thirteen patients underwent surgical resection followed by five cycles of induction chemotherapy and a single course of HDC with stem cell rescue. HS-II differed from HS-I with the addition of methotrexate to induction agents including cisplatin, etoposide, cytoxan, and vincristine. Consolidation chemotherapy included carboplatin, thiotepa, and etoposide. If the patient had no evidence of disease at the end of induction, regardless of second look surgery, then the patient proceeded to consolidation. If there was evidence of residual disease locally or with positive cerebrospinal cytology, consolidation was followed by RT. Four of 13 patients did not get all five induction courses, and one patient had no induction chemotherapy and proceeded straight to consolidation. One patient treated with HS-II who had only 1 cycle of induction died of *Staphylococcus aureus* meningitis. During induction there were eight episodes of bacterial sepsis among six HS-I patients, and all seven patients on HS-II had bacterial sepsis. Three of the 13 patients had fungal infections. Only two of the six HS-I patients and five of the seven HS-II patients went on to consolidation. RT was utilized in two HS-I and two HS-II patients. All six of the HS-I patients died of disease, but three of the seven HS-II patients were alive at the time of publication with no evidence of disease, and none had RT. There may have been a benefit to patients who received methotrexate, as all patients on HS-I died of their disease, whereas the reported EFS for HS-II was 43 ± 19% at 3 years. They also further supported that near total or GTR led to better outcomes than subtotal or partial resection with a significant difference in OS at 57 ± 18 versus 0%. The estimated EFS and OS at 3 years was 23 ± 11%. Although this analysis may show a possible benefit to the use of methotrexate and further supports better outcomes with more complete resection, the 3-year estimated EFS is comparable to that achieved with other therapeutic approaches.

A group from Italy reported eight patients treated on a clinical trial that included radiotherapy; ifosfamide, carboplatin, and etoposide (ICE); and HDC (Fidani et al., 2009). They had originally included cyclophosphamide, etoposide, carboplatin, and thiotepa, but after three of four patients had progressive disease on this therapy, they decided to exclude these courses and replace them with additional ICE. Only one of eight patients had metastatic disease at diagnosis, and complete resection was achieved in three patients. They defined overall survival as the time from the date of diagnosis until the date of death with a reported OS probability at 5 years of 50%. The group admits that the patient numbers were too small to make any real determination of survival compared with historic controls. Of the five patients who were reported as still alive at the time of publication, one did not proceed to HDC, one had relapse before HDC, one had relapse after HDC but was alive with salvage therapy, one proceeded through planned therapy including HDC and has no evidence of disease, and one was currently on therapy at the time of publication.

Researchers at The Hospital for Sick Children in Toronto reviewed their experience with HDC in ATRT from 2003 through 2008 and found six evaluable patients (Finkelstein-Shechter et al., 2010). All six patients had three cycles of carboplatin and thiotepa conditioning, although there were some differences in other therapies received by each patient. They reported four of six patients alive with no evidence of disease at a median follow up of 52 months, and three of the four patients did not receive any radiation. A team from the University of California, San Francisco, reported their experience with HDC in nine patients treated at their institution between 1997 and 2007 (Nicolaides et al., 2010). Patients had varied postsurgical therapy and conditioning regimens. They reported two patients alive with no evidence of disease after 78 and 98 months and one alive with disease after 19 months. Most recently a group from Korea enrolled nine patients on a prospective phase I/II trial to investigate tandem transplants in ATRT with course one including carboplatin, thiotepa, and etoposide and course two with cyclophosphamide and melphalan (Park et al., 2012). During therapy, five of the nine had progressive disease, and one patient died of disease. The remaining four were salvaged and preceded to tandem transplants. All patients on this trial were treated with the same conditioning regimen, which should make comparison easier, but of the five alive at the time of reporting, four had some deviation from therapy for salvage after progression. The protocol was written to allow for salvage therapies in the event of progressive disease while still allowing the patient to proceed to HDC. There was only one patient who proceeded through planned therapy including the post-transplant radiation without progressive disease. They reported a 3-year OS of 53.3 ± 17.3% and EFS of 0% and admitted that radiation may have been the most important component of therapy in the survivors.

No real conclusions can be made from published data regarding the role of high-dose chemotherapy in ATRT due to small patient numbers, multiple chemotherapy regimens, and additional salvage therapy that often includes radiation. The HS II protocol does point to increased survival with the addition of high-dose methotrexate to intensive chemotherapy, one course of high-dose chemotherapy and avoidance of radiation. This difference is likely due to additional systemic therapy targeted for better CNS penetration rather than the importance of continued inclusion of high-dose chemotherapy. Upfront intensive multimodal therapy for ATRT is needed, but the role of high-dose chemotherapy with autologous stem cell rescue remains unclear and its use should be balanced with overall toxicity of therapy.

## Investigating New Therapeutic Targets

The most often encountered genomic aberration in ATRT is monosomy 22 or a deletion or translocation of 22q11.2, which is best identified through fluorescence *in situ* hybridization (FISH; Biegel, 2006). Inactivating mutations of the *SMARCB1* gene (*hSNF5/INI-1*) at 22q11.2 is thought to be a crucial step in tumorigenesis (Biegel, 2006), but mutations can be identified in only about 76% of CNS ATRT tumor samples (Biegel et al., 2002). INI1 is one member of the BRG-associated factor (BAF) or SWI/SNF complex, which are important in chromatin remodeling (Biegel, 2006; Venneti et al., 2011). In 1998, Versteege et al. (1998) reported their work with 13 cell lines in which they identified the most frequent mutations in the hSNF5/INI1 gene. They compared their findings with corresponding primary tumor samples to verify that the mutations did not occur as a result of *in vitro* growth. The identified biallelic alterations corresponded with the “two-hit” model of oncogenesis. More recently a group has shown through whole-exome sequencing of 32 samples of rhabdoid tumors, which included 20 CNS tumors, that the overall mutation rate is relatively low in primary samples (Lee et al., 2012). The primary rhabdoid tumor genome was seen as relatively simple, but when three recurrent tumor samples were tested and compared to primary samples the mutation rate was increased eightfold. Other investigators at the time this genetic link became apparent were looking into the role of SWI/SNF complexes in cell cycle control. It was shown that complexes of activated versus inactivated retinoblastoma protein (Rb) with histone deacetylase (HDAC) and SWI/SNF controlled cell cycle progression through the G1 and S phases through the E2F pathway (Zhang et al., 2000). Using malignant rhabdoid cells, it was shown that ectopic expression of hSNF5/INI1 blocks entry into S phase, but this effect can be reversed by cyclin D1 or cyclin E (Versteege et al., 2002).

Since 1998, when the hSNF5/INI1 gene was identified as playing a role in MRTs, work to understand the mechanism that drives ATRT has provided potential therapeutic targets. Using the MON cell line, a group from Albert Einstein College of Medicine reintroduced INI1 and then analyzed a cDNA microarray to determine expression changes that may result in new therapeutic targets (Morozov et al., 2007). They found 63 genes that were upregulated and 18 that were downregulated. The majority of the downregulated genes were important in mitosis, including topoisomerase II alpha (TOP2A), aurora A (STK6), polo-like kinase (PLK), kinesin family member 2C (KIF2C), centromere protein F (CENPF), and pituitary-tumor transforming gene 1 (PTTG1). They found that interferon-stimulated genes were significantly increased early after the reintroduction of INI1. They then treated MON and STA-WT1 rhabdoid cell lines with interferon-alpha or -beta and found that, compared with controls, there were reduced cell numbers and increased flat cells 5–7 days after one round of replating. INI1 reintroduction also resulted in down-modulation of PLK1. With RNA interference, reduced PLK1 levels in rhabdoid cells resulted in reduced cell numbers, increased cell size, and altered morphology. The researchers concluded that drugs that induce interferons or target PLK1 or *cyclin D1* may be effective. There have been at least two phase I studies of PLK1 inhibitors (BI 2536 and BI 6727) in adults with solid tumors to date, and they both seem to be well tolerated with some element of hematologic toxicity in 15–30% of patients and at least stable disease in up to 40% of patients (Frost et al., 2012; Schoffski et al., 2012).

### Cyclin D1

Further investigation into the association of *INI1* and *cyclin D1* showed that loss of INI1 results in derepression of the transcription of *cyclin D1*, which may drive the cell through G1 cell cycle restriction. A group at Albert Einstein College of Medicine generated *Ini1*^+/−^ mice and showed that they have an increased incidence of rhabdoid tumors and there is a derepression of *cyclin D1*. They then crossed *Ini1*^+/−^ mice with *cyclin D1*^−/−^ mice and found that without *cyclin D1* expression, rhabdoid tumors failed to develop. When they reintroduced *INI1* into *Ini1*^−/−^ MON cell lines, they showed repression of cyclin D1 and activation of p16^INK4A^ (Tsikitis et al., 2005). A group at CHOP investigated 25 ATRT and 11 non-CNS MRT samples with confirmed *SMARCB1* loss. They aimed to correlate cell line and animal data with primary tumor samples, because there had been previous contradictions regarding the relationship between *SMARCB1*, *p16^INK4A^*, and *cyclin D1*. When staining for *p16^INK4A^*, they found that 17 of 25 (68%) of ATRT and four of 11 (36%) of non-CNS MRT were negative. They noted expression of *cyclin D1* in 20 of 25 (80%) of ATRT and 6 of 11 (54%) of non-CNS MRT (Venneti et al., 2011). These studies showed that *cyclin D1* may drive rhabdoid tumors and is expressed in a majority of primary ATRT tumor samples and may be an effective therapeutic target.

Research unrelated to ATRT has shown that HDAC inhibitors such as MS-275 or trichostatin A decrease the expression of cyclin D1 and can decrease cell proliferation in culture (Rosato et al., 2003; Hu and Colburn, 2005; Alao et al., 2006). Researchers at the University of Colorado at Denver used two ATRT cell lines and one primary short-term culture of a tumor sample to investigate the effects of three HDAC inhibitors: trichostatin A, suberoylanilide hydroxamic acid (SAHA), and SNDX-275 (Knipstein et al., 2012). They showed that all tested HDAC inhibitors decreased proliferation and that SNDX-275 increased the sensitivity of BT12 and BT16 cell lines to ionizing radiation. Retinoids such as all-trans retinoic acid have also been shown in the laboratory to inhibit cyclins and cyclin-dependent kinases, although the specific target of inhibition differs with the agent used (Kosaka et al., 2001). Other vitamin A analogs such as the rexinoid bexarotene have been shown to decrease the expression of cyclin D1, and this effect can be reversed by the HDAC inhibitor trichostatin A (Li et al., 2011). The induction of the transcriptional repressor DEC2 by bexarotene thus requires histone deacetylation as a method to suppress transcription of cyclin D1. Understanding these mechanisms of action further and the application of these methods to ATRT cells for further investigation may be warranted. Based on current information, the combination of a retinoid with an HDAC inhibitor should be avoided.

### Aurora A

The same group previously discussed from Albert Einstein College of Medicine also investigated the role of *Aurora A* in rhabdoid tumors (Lee et al., 2011). They used rhabdoid tumor cell lines MON, STA-WT1, and G401 and first showed that introduction of INI1 resulted in down-modulation of *Aurora A* by repression of gene promoter activity. When using si-Aurora A to down-modulate *Aurora A* in the cell lines, they saw a significant decrease in growth with enlarged cell morphology with 12–15% cell death in the treated cells and an increase in cleaved caspase 3 products. Using three primary human rhabdoid tumors, two primary mouse rhabdoid tumors, and a mouse xenograft derived from the human G401 cell line, they showed that all had a several-fold increase in Aurora A mRNA and all stained with α-Aurora A antibody. Later published data from the Pediatric Preclinical Testing Program (PPTP) showed intermediate to high response rates in rhabdoid mouse xenograft models treated with the Aurora A inhibitor MLN8237, with the most significant response seen in the KT12 cell line (Maris et al., 2010). MLN8237 is currently in use in clinical trials for adult and pediatric patients with varied tumors, including leukemia/lymphoma, melanoma, and solid tumors. There are many other aurora kinase inhibitors in various stages of clinical trials and with different specificities to Aurora A, B, or C that may be candidates for clinical trials in patients with ATRT (Dar et al., 2010).

### Insulin-like growth factor

Positive immunohistochemical staining with IGF-IR and its ligand IGF-II has been shown in two ATRT samples and suggests that an autocrine/paracrine loop is involved in ATRT (Ogino et al., 2001). Researchers from CHOP wanted to further investigate this pathway and first confirmed IGF-IR expression in eight formalin-fixed paraffin-embedded ATRT samples (D’Cunja et al., 2007). Through Western blotting, they showed IGF-IR was more highly expressed in ATRT than in normal brain, medulloblastoma, or glial tumors. BT12 and BT16 cells treated with IGF-IR antisense oligonucleotides decreased proliferation, increased apoptosis, and increased chemotherapeutic sensitivity to doxorubicin and cisplatin. Due to the interest in the IGF-I receptor signaling pathway, a single case of ATRT was evaluated in Poland for downstream effectors Akt or Erk as they relate to mTOR activation (Jozwiak et al., 2010). They found that Akt was upregulated while an inhibitor of Akt, PTEN, was not elevated compared with levels found in control brain. Members of the Erk cascade were not elevated in this sample. Others have shown that ATRT cell lines grown in serum-free media secrete insulin, supporting the autocrine/paracrine theory and that use of the IGF-IR inhibitor NVP-AEW541 inhibited proliferation and increased caspase 3 activation, although only at a high concentration (Arcaro et al., 2007). There are several preclinical studies and clinical trials related to the development of IGF inhibitors, whether through small molecule inhibitors such as nordihydroguaiaretic acid in prostate cancer (Ozkan, 2011; Friedlander et al., 2012) or breast cancer (Rowe et al., 2008) or receptor-inhibiting antibodies in multiple solid tumors (Adam et al., 2011).

### Tyrosine kinases

Multiple tyrosine kinase inhibitors are available, and they are often used for non-specific targeting of proliferative pathways in oncology. A group in Germany investigated 5 ATRT and 18 non-CNS MRT samples as well as two cell lines for expression of tyrosine kinases that are inhibited by imatinib (Koos et al., 2010). Previously, there was a report that the BT12 cell line had decreased proliferation after treatment with imatinib, although BT16 was not affected (Narendran et al., 2008). The German group found c-Abl staining in all 23 primary tumor samples as well as A204 and G401 cells. Imatinib as well as specific targeting of c-Abl with siRNA significantly reduced proliferation of both cell lines. The researchers commented on two ATRT patients treated with imatinib due to tumor expression of platelet-derived growth factor (PDGF) who did not achieve a response, and they hypothesized that this, as well as previous failure of response in the BT16 cell line, may both be related to c-Abl expression.

Three cell lines (BT12, BT16, and KCCF1) had dose-dependent inhibition of growth when exposed to sorafenib and sunitinib (Jayanthan et al., 2011). Analysis of the supernatant of cell culture media contained significant levels of PDGF and vascular endothelial growth factor. After showing that all three cell lines were affected by the tyrosine kinase inhibitors as well as irinotecan alone, the group showed that there are synergistic effects on cytotoxicity when used in combination. Others have reported that expression of epidermal growth factor receptor was absent in nine tumor samples of ATRT tested by FISH and immunohistochemistry (Jeibmann et al., 2006) and suggested that this may not be an effective target in ATRT.

**Figure 1:**
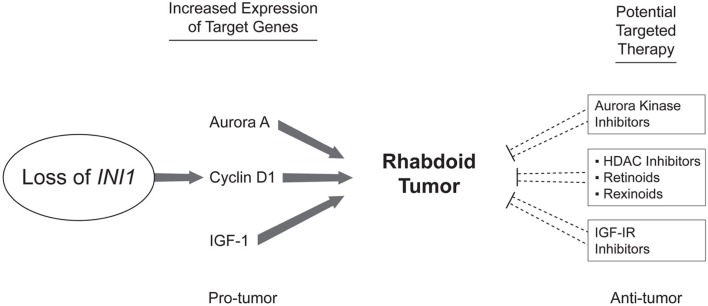
**Potential therapeutic targets in ATRT**. Studying the effects of the reintroduction of INI1 as well as the effects of its loss has led to the identification of multiple potential therapeutic targets. INI1 loss leads to increased cyclin D1 which propagates the cell through the G1-S checkpoint. HDAC inhibitors as well as Vitamin A analogs such as retinoids and rexinoids have been shown to inhibit cyclin D1. Aurora A signaling has also been shown to be important in ATRT and multiple inhibitors of Aurora kinase signaling are available. IGF-IR signaling may also play a role in ATRT and inhibitors are available for testing.

It is difficult to take early preclinical work and translate it quickly to the clinical realm but this is desperately needed in a tumor such as ATRT with such dismal outcomes. Newer methods of high throughput screening and “clinics” of experimental animals have sped the identification of new effective drugs but again may not translate into clinical effectiveness. As is always a concern with preclinical data the use of cultured cell lines may not accurately represent the biology of the primary disease but is a cost effective means to new target identification. As discussed above, PLK1 levels have only been investigated in a single cell line and thus this target has the weakest evidence for proceeding further with clinical applications. Further work is needed with primary tumor samples, primary cell culture, and animal models. Aurora kinase A has the most compelling preclinical data as investigations have included cell line, primary tumor, and mouse xenograft models and some investigators have proceeded with clinical trials using MLN8237 for ATRT. Cyclin D1 is the target with the second most interesting preclinical data with cell line work in only a single cell line but further investigations included a mouse model showing that cyclin D1 seems necessary for tumor formation. This was further validated by CHOP with 80% cyclin D1 expression of 25 ATRT primary tumor samples. HDAC inhibitors and retinoids have been in used in pediatric oncology for many years and may be quickly applied to ATRT although more specific inhibitors of cyclin D1 would give better therapeutic information. See Figure 1 for a summary of the current targets identified in this review.

## Conclusion

ATRT is an aggressive malignancy with poor survival especially in patients with metastatic disease and in those who are younger than 3 years old at diagnosis. Multiple therapeutic approaches have been attempted over the last two decades in an attempt to increase survival in these patients without much success. RT seems to be the most important component of therapy but is often not an option. Survival seems to be better with focal radiation, and there may be a role for intrathecal chemotherapy in patients who are not candidates for radiation. There is no accepted standard chemotherapy, but intensive alkylator-based chemotherapy regimens, regimens with high-dose methotrexate, and regimens that include HDC with stem cell rescue may be more effective in these patients. Efforts to delay radiation often fail, and most reported cases of survivors, even when including HDC with stem cell rescue, frequently require repeat surgery and radiation.

More potential therapeutic targets have become apparent as we learn more about the biological mechanisms that drive tumor formation and proliferation. Cell line data and preclinical mouse models are not ideal but are necessary to determine the effectiveness of chemotherapy and small molecule inhibitors. It is unlikely that any of these agents alone will result in increased survival, and they must be combined with current intensive therapy if they are expected to have any lasting effects. Combinations of these targeted agents will also likely be required, just as our current therapy includes chemotherapeutics with different targets to affect cell cycle progression.

More rapid progression of these agents from the laboratory to the clinic is needed. Many agents described in this review are already in clinical use but have not had reported use in ATRT, and these agents may be able to move forward more quickly into clinical trials. Current therapy seems to be reaching the maximum levels of tolerable intensification without bringing a significant change in outcomes, and new approaches are desperately needed to advance therapy.

## Conflict of Interest Statement

The authors declare that the research was conducted in the absence of any commercial or financial relationships that could be construed as a potential conflict of interest.
